# Non-progressive breast carcinomas detected at mammography screening: a population study

**DOI:** 10.1186/s13058-023-01682-9

**Published:** 2023-07-04

**Authors:** Torunn Heggland, Lars Johan Vatten, Signe Opdahl, Harald Weedon-Fekjær

**Affiliations:** 1 Oslo Centre for Biostatistics and Epidemiology [OCBE], Research Support Services, Oslo University Hospital, Oslo, Norway; 2 Oslo Centre for Biostatistics and Epidemiology, Department of Biostatistics, Institute of Basic Medical Sciences, University of Oslo, Oslo, Norway; 3 Department of Public Health and Nursing, Faculty of Medicine and Health Science, Norwegian University of Science and Technology, Trondheim, Norway

**Keywords:** Mammography screening, Non-progressive breast carcinoma, Observation study, Age-period-cohort model

## Abstract

**Background:**

Some breast carcinomas detected at screening, especially ductal carcinoma in situ, may have limited potential for progression to symptomatic disease. To determine non-progression is a challenge, but if all screening-detected breast tumors eventually reach a clinical stage, the cumulative incidence at a reasonably high age would be similar for women with or without screening, conditional on the women being alive.

**Methods:**

Using high-quality population data with 24 years of follow-up from the gradually introduced BreastScreen Norway program, we studied whether all breast carcinomas detected at mammography screening 50–69 years of age would progress to clinical symptoms within 85 years of age. First, we estimated the incidence rates of breast carcinomas by age in scenarios with or without screening, based on an extended age-period-cohort incidence model. Next, we estimated the frequency of non-progressive tumors among screening-detected cases, by calculating the difference in the cumulative rate of breast carcinomas between the screening and non-screening scenarios at 85 years of age.

**Results:**

Among women who attended BreastScreen Norway from the age of 50 to 69 years, we estimated that 1.1% of the participants were diagnosed with a breast carcinoma without the potential to progress to symptomatic disease by 85 years of age. This proportion of potentially non-progressive tumors corresponded to 15.7% [95% CI 3.3, 27.1] of breast carcinomas detected at screening.

**Conclusions:**

Our findings suggest that nearly one in six breast carcinomas detected at screening may be non-progressive.

**Supplementary Information:**

The online version contains supplementary material available at 10.1186/s13058-023-01682-9.

## Background

Breast cancer develops gradually from early preclinical stages to a stage that causes clinical signs and symptoms. Mammography screening programs aim to detect breast carcinomas in their preclinical detectable phase, and to reduce breast cancer mortality through earlier treatment. Some carcinomas may progress rapidly into a clinical stage, whereas others may progress slowly and remain in the preclinical phase for a long time period [1]. As almost all diagnosed breast carcinomas undergo treatment, their evolution from a preclinical to a clinical phase is not directly observable. Nevertheless, it has been suggested that a certain proportion of preclinical carcinomas do not have the potential to reach a clinical stage. Previous studies have provided a wide range of estimates for preclinically detectable carcinomas without a clinical potential, ranging from 1 to 42% [2–6]. Although this phenomenon cannot be studied directly, indirect methods may be used to indicate the proportion of carcinomas that may not progress to clinical disease within a reasonable time. The non-progressive cases may account for a substantial proportion of the over-diagnosis of breast cancer that may be attributed to mammography screening [7].

However, if all screening-detected breast carcinomas have a clinical potential**,** the long-term cumulative incidence of breast carcinoma, with or without screening, would be similar, conditional on the women being alive. On the other hand, if an excess cumulative incidence at a high age is associated with screening, that excess would indicate carcinomas without a clinical potential.

We used high quality data from the Norwegian national mammography screening program (BreastScreen Norway) to estimate the proportion of screening-detected breast carcinomas without a potential to develop into clinical disease by 85 years of age in the absence of screening.

## Methods

### Study population and data sources

The study included all women aged 49–84 residing in Norway during the period 1987–2019, and the associated first cases of invasive breast cancer or ductal carcinoma in situ (DCIS). The Cancer Registry of Norway provided the data aggregated by the women’s birth cohort, calendar year and county of residence.

Reporting to the Cancer Registry of Norway is mandatory, and diagnostic information is obtained separately from clinicians, pathologists, and death certificates, with only 0.2% of all cancers ascertained from death certificates alone [8]. Reporting of DCIS became mandatory from 1993, but the stable DCIS incidence rates around 1993 suggest that the registration was nearly complete also before mandatory registration (Additional file 1: Fig. S1).

BreastScreen Norway, administered by the Cancer Registry of Norway, targets women 50–69 years of age in biennial mammography screening. The program was initiated in four large counties (approximately 40% of eligible women in Norway) in late 1995 and early 1996, and was rolled out in the remaining 14 Norwegian counties during the period 1999–2005 (Table 1). Women are invited according to birth cohort to county-wise screening rounds, and we used the exact start and end dates of screening invitations for the respective birth cohorts in each county. The overall attendance to the program has been relatively stable at around 76% [9]. Full-field digital mammograms gradually replaced screen film mammograms from the year 2000 [10].

**Table 1:** The BreastScreen Norway mammography screening program and women included in the study

*Study data*	
Area	Norway
Time period	1987–2019
Age range^a^	49–84
Design	Dynamic cohort study
Person-years under study	23,709,444
DCIS or invasive breast cancer cases	63,378
*BreastScreen Norway*	
Implementation period	1995–2005
Target age range and frequency	50–69 every second year
Attendance rate	76%
Screening test	Two-view mammography
Evaluation	Two independent readers
Women in invited birth cohorts	1,063,409 (approximated)
Proportion of cases screening-detected aged 50–69 years	68% (year 2016–2019)

^a^Data are given by birth cohort and calendar year, aiming at 49–84 years of age

Menopausal hormone therapy is associated with increased risk of breast cancer [11], and was extensively used in Norway during the period coinciding with the first introductions of screening [12]. However, its use declined sharply from around 2002 after hormone therapy was linked to an increased risk of cardiovascular disease [13]. To avoid potential biases by variation in the use of hormone therapy, we applied county-specific sales figures from the Norwegian Drug Wholesales Statistics of preparations containing both estrogen and estrogen-progestogen combinations, scaled according to the age distribution among women with a prescription for hormone therapy in the Prescription Database of Norway. Both sources are available at the Norwegian Institute of Public Health (See Additional file 1).

### Statistical analysis

To estimate the frequency of non-progressive breast carcinomas, we followed women up to 85 years of age, and compared the cumulative rate of breast carcinoma between screened women and women without screening. In the lack of a randomized non-invited comparison group, and since no birth cohorts invited to screening from age 50 have yet reached the age of 85 years, we took advantage of the gradual county-wise introduction of BreastScreen Norway to distinguish the effects of screening by age from temporary incidence changes (Additional file 1: Fig. S2). Using the Norwegian data, we estimated likely age-specific incidence rates in the presence and absence of screening, where the cumulative rate is the sum of the age-specific incidence rates.

We modeled the incidence of first cases of breast carcinoma among Norwegian women using an extended Age-Period-Cohort (APC) Poisson regression model [14, 15], similar to our previous modeling work [12, 16]. The gradual county-wise introduction of BreastScreen Norway provided data with different screening program status at the same calendar times, which could be exploited in the APC Poisson regression model. We included variables for different effects of the screening program. For the screening period, we added separate variables for the initial screening round at 50–51 years of age, for the second screening round at 52–53 years of age, and for the subsequent eight screening rounds, modeled as the proportion of the calendar year women in a birth cohort were covered by the specific parts of the program. The separate variable for the second screening round was added to account for cases that might have been overlooked at the initial screening. We applied natural cubic splines to allow for nonlinear effects by age for the screening period (inner knot at age: 60). After the screening period, we allowed for a gradual declining effect of previous screening by applying natural cubic splines for the time since screening cessation (inner knots at year: 1, 2, 5, 10, and 13) in addition to a variable for no longer being covered by the screening program.

Since changes in incidence typically appear gradually [17], we also used natural cubic splines to smooth the effects of age (inner knots at age: 50, 52, 54, 56, 60, 70, and 80), period (inner knots at year: 1997 and 2009) and birth cohort (inner knots at birth cohort: 1929 and 1943), in order to limit the number of variables in the model. For the age component, we added more knots around the age of menopause to increase modeling flexibility for ages at which the effects of certain breast cancer risk factors tend to change [18]. Each county was assigned its own breast carcinoma incidence level. As in our previous modeling of breast cancer incidence, we applied a one-year time lag for the hormone therapy variable [12] to reflect the likely time lag between use of hormone therapy and increased risk of breast carcinoma [11]. Other changes in risk factors were taken into account by the age, period and cohort components.

The women contributed person-years until they were censored due to death or end of follow-up. At screening implementation, women in the entire target age range of 50–69 years were invited to their first screening. To limit model complexity, data regarding initial or second screening at higher ages, above the age of 53 or 55 years, respectively, were not used in the modeling. It was thus assumed that the relative risk of breast carcinoma from the third screening round onwards, and for post-screening women, was similar to the incidence for women screened since the age of 50. The full specification of the incidence model is given in the Additional file 1.

We calculated the average estimated age-specific incidence rates across all Norwegian counties, both in the presence and absence of invitations to the full screening program, by applying the incidence model on calendar year 2019 for the 1969 birth cohort, with hormone therapy set at the national 2019 level.

We estimated the frequency of non-progressive screening-detected breast carcinomas as the difference in the cumulative rates at 85 years of age between screening and non-screening scenarios. The age of 85 years was chosen to allow for disease progression within a reasonable time (15 years), while still having a substantial number of person years under study.

To estimate the proportion of non-progressive cases related to screening, we calculated the frequency of screening-detected breast carcinomas from the APC model. We multiplied the cumulative rate for ages 50–69 years in the presence of screening with the observed proportion of screening-detected carcinomas among women 50–69 years of age in the years 2016–2019 (Table 1, Additional file 1: Fig. S4).

To facilitate comparison with other studies, we performed analyses without inclusion of DCIS. Active treatment of screening-detected DCIS might have prevented the transition of DCIS to invasive cancer [19]. Thus, to estimate invasive non-progressive cases only, using population data, will likely lead to a result biased toward the null.

To assess statistical uncertainty, we calculated 95% bootstrap percentile confidence intervals (CI) based on 10,000 repetitions. For sensitivity analyses we either removed the hormone variable, the period variable or the interaction between age and subsequent screening. As a sensitivity analysis, we further extended follow-up to 90 and 95 years of age, under the conservative assumption that the difference in age-specific incidence at 85 years of age between the screening and non-screening scenario remained constant at higher ages.

All statistical analyses were conducted using the R statistical package (version 4.2.2, R Foundation for Statistical Computing, Vienna, Austria) [20].

## Results

The study included 63 378 cases of invasive breast cancer or DCIS among 23,709,444 person-years of observation (Table 1).

**Fig. 1:**
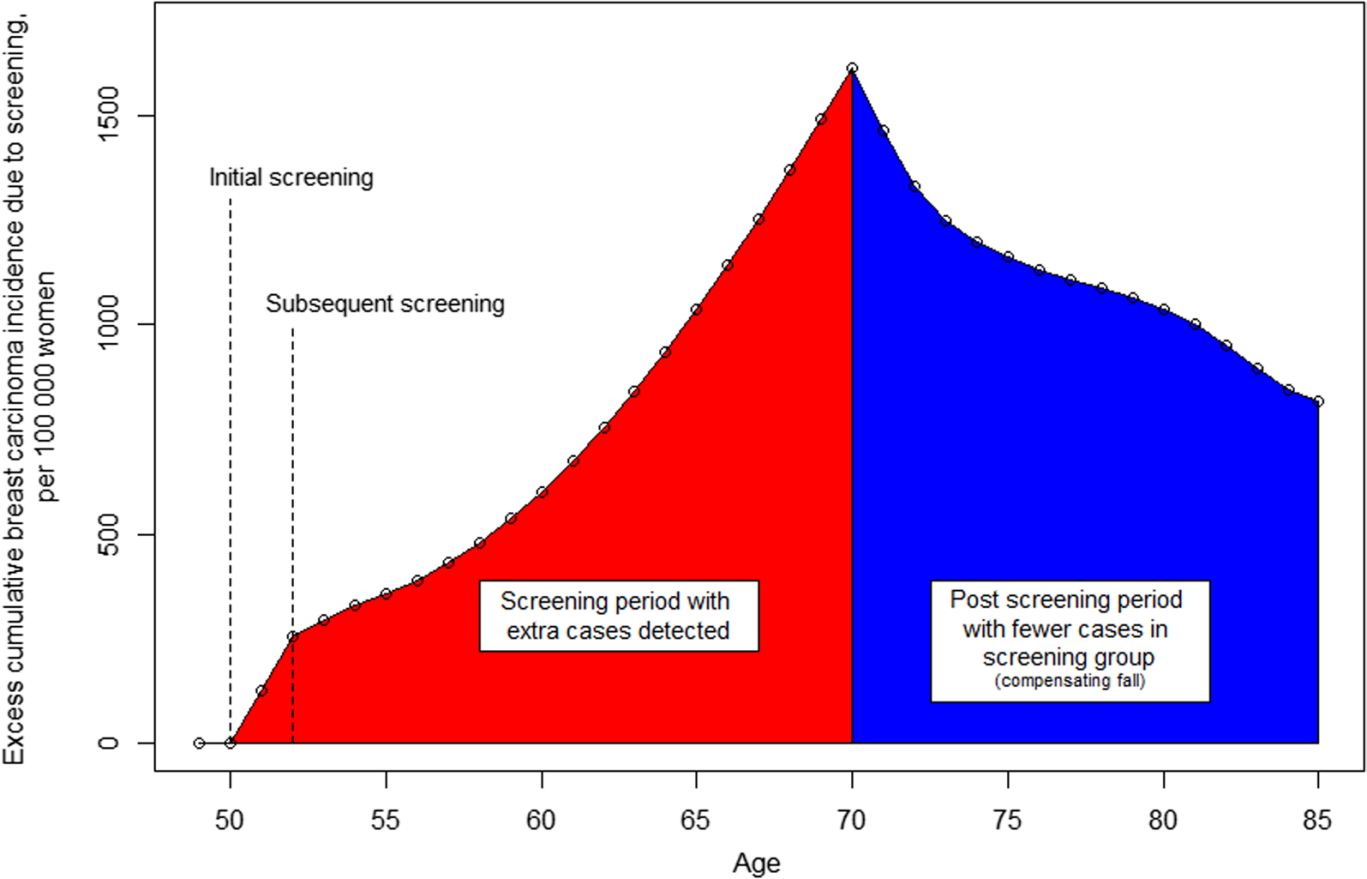
Excess in cumulative incidence of breast carcinomas per 100,000 women in the presence of screening versus absence of screening (all numbers conditional on the women being alive)

The excess frequency of breast carcinomas related to screening increased gradually from the start of screening at around 50 years of age until the invitations ceased by age 70, followed by a gradual decrease after screening cessation (Fig. 1). After the first screening, we observed an excess of 256 cases per 100,000 women invited to screening compared to women who were not invited. The excess over the screening period had increased to 1 614 cases per 100,000 women when screening ceased at 70 years of age (Table 2). At 85 years of age, the excess frequency had decreased to 814 cases (per 100,000 women invited to 10 screening rounds), implying that these cases were detected at screening and would not have reached a clinical stage by 85 years of age. In other words, around 50% of the excess incidence detected during the screening period (50–69 years) would have appeared as a clinical cancer by 85 years of age in women who were not invited to screening, conditional on the women being alive at that age (Fig. 1, Table 2).

**Table 2:** Basis for excess cumulative incidence calculations, based on estimates from the APC incidence model calculated for the 1969 birth cohort in 2019

Age	Relative incidence with screening program 50–69 years of age	Among 100,000 women		
		Excess incidence	Incidence deficit	Excess cumulative incidence
50	1.58	128	–	128
51	1.58	128	–	256
52	1.17	37	–	293
53	1.17	37	–	330
54	1.11	26	–	356
55	1.14	33	–	389
56	1.16	41	–	430
57	1.19	49	–	479
58	1.21	57	–	536
59	1.23	65	–	601
60	1.25	73	–	674
61	1.26	80	–	754
62	1.27	87	–	841
63	1.28	94	–	935
64	1.29	100	–	1035
65	1.29	106	–	1141
66	1.29	112	–	1253
67	1.29	117	–	1370
68	1.29	121	–	1490
69	1.29	124	–	1614
70	0.66	–	− 151	1463
71	0.71	–	− 132	1331
72	0.83	–	− 83	1248
73	0.89	–	− 52	1197
74	0.93	–	− 37	1160
75	0.94	–	− 30	1131
76	0.95	–	− 24	1107
77	0.96	–	− 21	1085
78	0.96	–	− 22	1063
79	0.95	–	− 27	1036
80	0.93	–	− 37	999
81	0.92	–	− 49	950
82	0.91	–	− 55	895
83	0.92	–	− 50	845
84	0.95	–	− 31	814

All numbers conditional on the women being alive

**Fig. 2:**
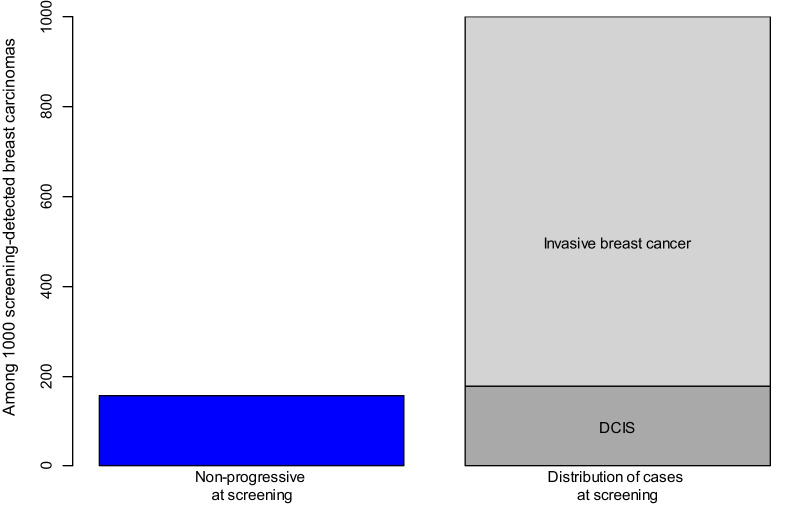
Estimated non-progressive cases among 1000 screening-detected breast carcinomas, and the observed proportions of DCIS and of invasive breast cancer among screening-detected lesions in BreastScreen Norway for the period 2016–2019

We estimated that 15.7% [95% CI 3.3–27.1] of screening-detected breast carcinomas do not have the potential to reach a clinical stage before 85 years of age (Table 3, Fig. 2). Among women invited to 10 screening rounds we found that 0.8% were likely to be diagnosed with a non-progressive breast carcinoma, and this corresponds to one non-progressive case among 123 women invited to 10 screening rounds. By taking the attendance rate to the screening program into account, we estimated that 1.1% (0.8%/0.76) of regularly participating women would be diagnosed with a non-progressive breast carcinoma during the course of 10 screening rounds.

**Table 3:** Non-progressive breast carcinomas, with follow-up until 85 years of age

Model	Proportion non-progressive carcinomas of screening-detected cases	Probability of a non-progressive carcinoma after 10 screening rounds	Number of women screened for 10 rounds per screening-detected non-progressive carcinoma	AIC^a^
Main estimate	15·7% (3.3,27.1)	0·8% (0.2,1.4)	123 (69,491)	72,843
*Sensitivity analysis: (modifications of applied model)*				
Without age-screening interaction	9·4% (− 2.8,20.9)	0·5% (− 0.1,1.1)	209 (− 1162,1805)	72,849
Without hormones	16·5% (4.8,27.5)	0·8% (0.2, 1.3)	130 (76,429)	72,933
Without period component	15·9% (4.4,27.1)	0·8% (0.2,1.4)	122 (71,411)	72,846

^a^Lower AIC indicates a better model fit

Without the inclusion of DCIS cases, we estimated that 5.2% [95% CI − 9.0 to 17.4] of screening-detected invasive breast cancers are not likely to reach a clinical stage before 85 years of age (Additional file 1: Table S4).

Sensitivity analyses indicated an adequately robust estimation model, where the estimated proportion of non-progressive cases ranged between 9.4 and 16.5% (Table 3). The largest difference was seen after removing the interaction term between age and screening. However, the model fit decreased when that term was removed, supporting to keep the interaction in the model since sojourn time may increase by age [21]. Our applied incidence model fitted the data better than any sensitivity analyses, measured by Akaike’s information criteria (AIC). If we assume that the observed difference in age-specific incidence at 85 years of age remains constant beyond that age, we estimated that 12.7% and 9.7% of screening-detected cases do not have the potential to reach a clinical stage by 90 and 95 years of age, respectively.

## Discussion

We estimated that 15.7% of screening-detected breast carcinomas are likely to be non-progressive, based on follow-up of cases until 85 years of age. The estimate is for Norwegian women attending public screening, but is likely to be relevant for other populations undergoing biennial screening in similar age groups.

In this study, we included both invasive breast cancers and pre-invasive DCIS as transitions from DCIS to invasive breast cancer cannot be directly observed. Unfortunately, non-progressive DCIS cannot be reliably separated from non-progressive invasive breast cancer in a population study, without adding uncertain biological assumptions.

Before mammography screening, DCIS was less common, and its clinical fate is still largely unclear [22]. A recent review indicated that progression to invasive breast cancer may occur for only one in six detected DCIS lesions [19]. As the DCIS cancerous cells are confined to the milk ducts and have not yet penetrated the ductal basement membrane, DCIS can be seen as an early stage breast carcinoma. It is possible that invasive breast cancers are generally preceded by a non-observable DCIS stage [23], and DCIS may be a plausible candidate for a fairly large proportion of non-progressive cases. Our estimated proportion of non-progressive carcinomas associated with screening was lower than that of the DCIS proportion on screening (Fig. 2), with DCIS comprising 18% of breast carcinomas detected in the screening program during 2016–2019. This supports that DCIS may constitute a large fraction of non-progressive carcinomas. Some countries have a lower proportion of screening-detected DCIS than is registered in Norway, whereas others have a higher proportion [24], and these differences may influence the estimated level of non-progressive cases.

Following the introduction of the screening program, the age of previously invited women gradually increased. In order to distinguish the effects of previous screening on breast carcinoma incidence for the entire age range 70–85, long-term follow-up is necessary. Even with our 24 years of observation after the initiation of BreastScreen Norway, a limited number of birth cohorts who were invited to screening had reached the age of 80 years during our follow-up. This increases the study’s statistical uncertainty, illustrated by the quite wide bootstrap confidence intervals for the non-progressive cases detected at screening.

Follow-up beyond the age of 85 years could have reduced the non-progressive proportion of screening-detected carcinomas, because it is not known whether excess cases related to screening have stabilized at the age of 85 (Fig. 1). It is a rather strong assumption that differences in incidence between screened and unscreened cohorts are constant at old age, and the results of extending follow up to 90 or 95 years should therefore be interpreted with caution.

It may seem surprising that as much as 50% of the excess cases in screened age groups had not been detected by the age of 85 years without screening, whereas the corresponding non-progressive proportion was only 15.7% among screening-detected cases. This difference reflects, however, that the screening-induced excess cases only make up a smaller fraction of the screening-detected cases.

The transition from analogous to digital screening technology during the observation period could be a source of bias in the analysis, due to increasing screening test sensitivity associated with the digital technology [25]. A higher sensitivity would immediately affect ongoing screening, while an increased compensatory drop in incidence after cessation of screening may only appear after some time. Possibly, this could result in a slightly higher estimate of the non-progressive cases.

Also, a substantial proportion of women reported having mammograms prior to attending BreastScreen Norway, but it is uncertain how much of this was screening mammography [26]. Population data show no clear increase in breast cancer incidence before the screening program started [27], indicating limited pre-screening opportunistic screening. Opportunistic screening among women who are no longer targeted by the program could also have increased our estimate of non-progression somewhat, but most opportunistic screening likely ceased well before the age of 85 years.

## Comparison with other studies

Our estimate of non-progression is lower than that of some earlier studies (2–4). While the original models of the natural history of breast cancer assumed that all are progressive [28–30], this assumption has been challenged. The Wisconsin Breast Cancer Epidemiology Simulation Model estimated that around 42% of breast carcinomas may have a limited malignant potential, meaning they regress or become undetectable by screening unless they are detected within two years of reaching full size [2, 31]. Using data from the first screened Norwegian counties, Westvik et al. [32] demonstrated that models with only progressive tumors cannot fully explain the incidence level of invasive breast cancer that was observed after the introduction of screening. Utilizing more data, we estimated fewer non-progressive cases than indicated in that study. Zahl et al. [3] compared age-matched Norwegian cohorts with different screening exposure and found 22% higher cumulative incidence of invasive breast cancer in a cohort that was invited three times during a 6-year period, compared to a cohort that was invited only once at the end of an earlier, partially overlapping, 6-year period. This implied that 18% of invasive cases in the frequently screened group were not detected in the group with a single mammogram. However, high use of menopausal hormone therapy at the time when screening was introduced could have caused an increase in breast cancer risk [33]. To avoid that problem, Zahl et al. [4] conducted a similar study in Sweden for a time period with less hormone therapy, and the result of that study implied that 12% of invasive cases in the frequently screened group were not detected in the group with a single mammogram. To explain the findings, the researchers suggested that some invasive cancers could have regressed.

The lower estimate of non-progressive breast carcinomas that we found compared to other studies may be attributed to a more comprehensive modeling of age and cohort trends. On the other hand, several studies estimated lower levels of non-progressive tumors than we report in the present study [5, 6, 34]. These studies typically applied specific assumptions regarding tumor progression, as given by Markov models in Tan et al. [34] and Wu et al. [5]. Tan et al. [34] used data from the Östergötland randomized controlled trial (1978–1984), and estimated that 91% of breast lesions were aggressive, meaning that 9% of lesions may be harmless. Wu et al. [5] used screening data (1989–2014) from the population-based screening program in Stockholm, and estimated that 0.54% of screening-detected breast carcinomas may be non-progressive. However, screening at private clinics and in the Stockholm trial may have misclassified some screening-detected cases, leading to underestimation of the non-progressive proportion [5]. Using an approximate Bayesian simulation model fitted to French data (1991–2006), Seigneurin et al. [6] estimated that 7–8% of screening-detected cases may be non-progressive. Ryser et al. [7], also using a Bayesian approach, estimated that 6.1% of screen-detected cases may be indolent in a program of biennial screening from 50 to 74 years of age. Overall, these studies in combination with our estimates, suggest that some screening-detected cases may be non-progressive, or alternatively, they may reach a clinical stage at a very old age. The findings indicate a need for a better understanding of breast carcinoma progression.

## Conclusions

We found that breast carcinomas not progressing to clinical cancers by 85 years of age might comprise a substantial proportion of screening-detected cases. Better knowledge of tumor progression is needed to optimize treatment of screening-detected breast carcinomas.

## Supplementary Information

**Additional file 1**. APC model specification.

## Data Availability

The data are available for research projects from the legal administrator of the data, the Cancer Registry of Norway. The interpretation and reporting of these data are the sole responsibility of the authors, and no endorsement by the Cancer Registry of Norway is intended nor should be inferred. For data requests use Datautlevering@kreftregisteret.no.

## Abbreviations

DCIS: Ductal carcinoma in situ
APC model: Age-period-cohort model
CI: Confidence interval
