# Supplementary material for: Non-progressive breast carcinomas detected at mammography screening: a population study
Source: Breast Cancer Res. 2023 Jul 4;25:80. doi: 10.1186/s13058-023-01682-9 (PMC10318793; doi:10.1186/s13058-023-01682-9)
Supplement: Supplementary file 1 — Additional file 1. APC model specification. [file 13058_2023_1682_MOESM1_ESM.pdf]

Additional file 1

Registration of DCIS 1
Screening data for the modelling 2
Supplementary information regarding the hormone therapy variable 2
The extended APC Poisson regression model for breast carcinoma incidence 3
Calculating the proportion of breast carcinomas that are screening-detected 6
Analysis of invasive breast cancer only 6
References 8

Registration of DCIS

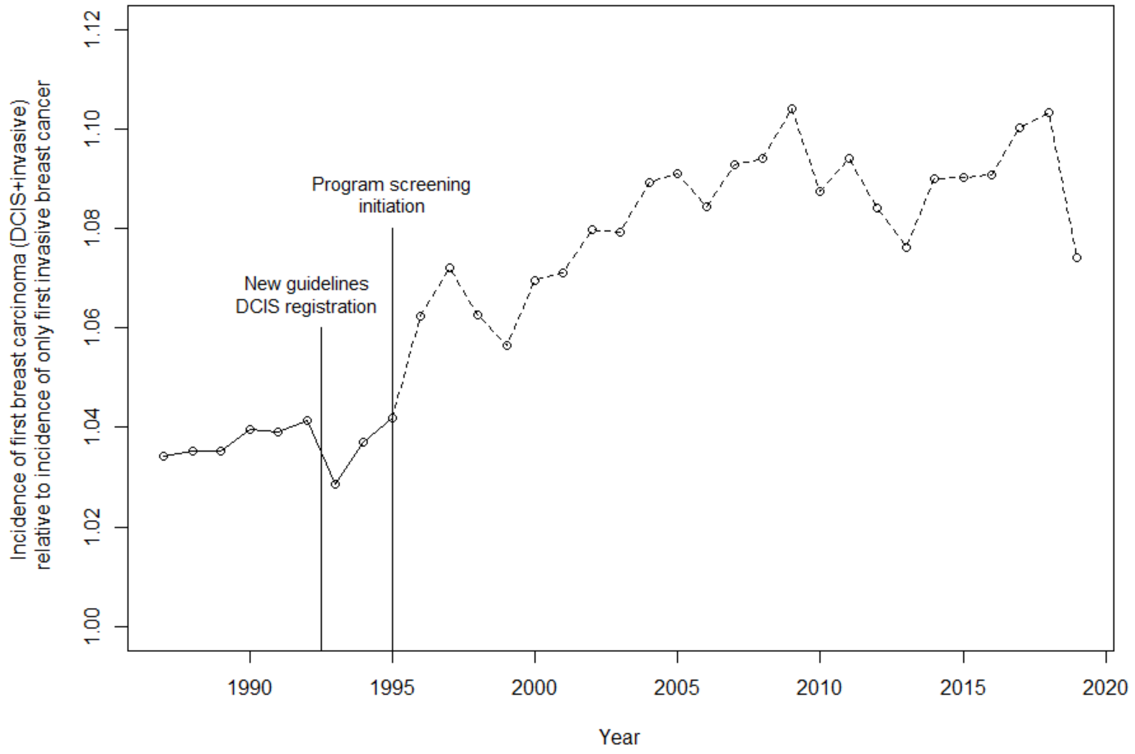

Figure S.1 First cases of DCIS or invasive breast cancer relative to first cases of only invasive breast cancer, for all ages in Norway.

Screening data for the modelling

To estimate screening effects, we take advantage of the gradual Norwegian country wise screening introduction.When screening starts in a county, the entire age range 50-69 is invited to screening for the first time. Later screening rounds will have both invitations to initial screening for the youngest women, and invitations to continued screening for the other targeted women. The number of earlier screening women gradually increase, and the age range for previously screened women increases with time (Figure S.2a). The gradual implementation of BreastScreen Norway (Figure S.2b) creates many contrasts in incidences between counties with different screening status, which is used to estimate the effects of screening. In the modelling, we excluded data for the initial and second screening round for women aged above 53 years when invited to screening for the first time, as this is atypical data not used in our final calculations regarding the effects of the fully implemented program.

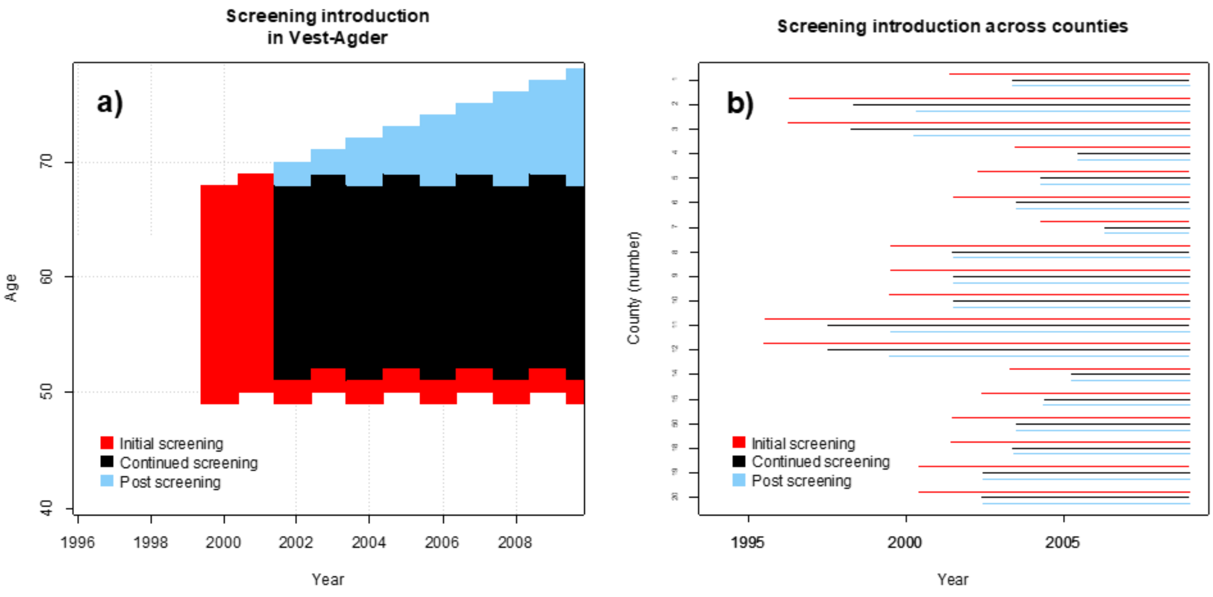

Figure S.2 a) Lexis diagram showing the screening introduction in one example county (Vest-Agder) b) Diagram showing screening status in each county by time.

Supplementary information regarding the hormone therapy variable

The national sales statistics of hormone therapy preparations have been available electronically since 1987. We here used data on both estrogen and estrogen-progestogen combinations. This equals, G03C and G03F, respectively, according to the Anatomical Therapeutic Chemical classification (1). County-specific sales statistics have been available in electronic form in four of the counties since 1992, and since 1999 for all counties. For the years without electronically available county-specific sales statistics, we used statistics collected from paper lists for 1987, 1991, and 1995, combined with linearly interpolated proportions of national use for the years in between.

The Prescription Database of Norway is available from 2004. For the period 1987-2003, we used the age distribution of prescription users in 2004 as an approximation. Among prescriptions in the Prescription Database of Norway, we found that a small proportion of hormone therapy was prescribed to men. When calculating the hormone therapy variable, we accounted for this use among men.

The extended APC Poisson regression model for breast carcinoma incidence

The breast carcinoma incidence model is given by

$$R_{c,p,r} = exp(A_a + ns(A_a, kn_a) + ns(P_p, kn_p) + ns(C_c, kn_c) + S_1 * scr_1 + S_2 * scr_2 + (S_{3+} * scr_{3+} + ns(A_a, kn_{A_a * scr_{3+}}) * scr_{3+}) + (S_{pr} * scr_{pr} + ns\left(timeScr_{pr}, kn_{scr_{pr}}\right) * scr_{pr}) + H * ht + County_r)$$

where $R_{c,p,r}$ is the breast cancer incidence rate in birth cohort c at period p for county (region) r, A_a is the age component for age a, P_p is the period component for period p, C_c is the cohort component for birth cohort c, and $ns(\dots)$ denotes the natural cubic splines functions. For the age component we specified inner spline knots at age 50, 52, 54, 56, 60, 70 and 80 years (kn_a). We used three degrees of freedom for the period component, and for the birth cohort component, both with knots set at the corresponding quantiles (kn_p and kn_c, respectively). Further S_1, S_2, S_{3+} and S_{pr} are coefficients for scr_1, scr_2, scr_{3+} and scr_{pr}, respectively, reflecting initial screening, second screening, continued screening and previous screening. For the natural cubic splines applied to the age component for subsequent screening rounds, we specified an inner spline knot at 60 years of age, and boundary knots at ages 53 and 72 years ($kn_{A_a * scr_{3+}}$). $timeScr_{pr}$ is the time since screening cessation (mean value over each calendar year), and we specified inner spline knots at 1, 2, 5, 10 and 13 years ($kn_{scr_{pr}}$). H is the coefficient for the hormone therapy variable ht. Each county was assigned its own level, as given by the $County$ variable, while the logarithm of the number of person-years under study was used as offset to adjust for variations in person-years.

For some Age-Period-Cohort combinations we use proportions of the person years under screening in the modelling. Here the applied formula becomes an approximation, but a highly accurate approximation within our relevant parameter space with likely only negligible bias.

Remark on the use of proportions in the applied Poison regression model

The watchful reader might notice that the use of proportions in our Poisson regression formula is an approximation. If we have a screening variable with coefficient β that is present among p of the observed persons-years, the expected rate across all the person-years becomes:

$$R_{precise} = (1 - p) * \exp(\alpha) + \mathrm{p} * \exp(\alpha + \beta)$$

By which:

$$R_{approx} = exp(\alpha + \beta * p)$$

is an approximation.

As shown by Duffy et al. 2007 (2), the bias can be considerable for very large relative risks. The approximation works, however, very well for small and moderate β values. This as both $R_{precise}(p = 0) = R_{approx}(p = 0)$, $R_{precise}(p = 1) = R_{approx}(p = 1)$, and the expontinal growth of R_{approx} is almost linear for moderate β and $0 \leq p \leq 1$ (Figure S.3). In our setting, most indicators were zero or one, and the largest relative risk was 1.6, supporting little bias.

Simulating the use of screening indicators given as exposed proportion, we find that the bias is probably negligible for our observed relative risks, with maximum biases of 0.2% (Table S.1).

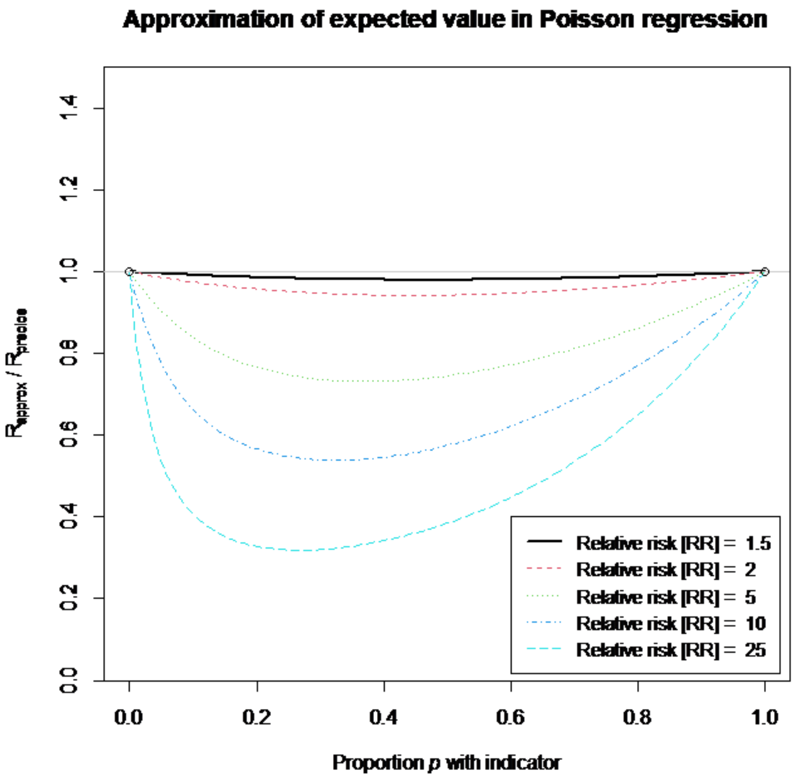

Figure S.3 The effect of using proportions directly in Poison regression; comparison of approximated vs. true expected values.

Table S.1 Simulations of estimating bias when using screening indicators directly as proportions in Poisson regression. All calculations is based on 1 000 000 simulations.

	Screening start	Proportions exposed	Median bias %
**Screening intro, relative risk 1.6**	January 1.	0 , 0 , 1 ,1 , 0	0.0 %
	April 1.	0 , 0 , $\frac{3}{4}$, 1 , $\frac{1}{4}$	+ 0.1 %
	July 1.	0 , 0 , $\frac{1}{2}$, 1 , $\frac{1}{2}$	+ 0.3 %
	October 1.	0 , 0 , $\frac{1}{4}$, 1 , $\frac{3}{4}$	+ 0.1 %
**Leaving screening, relative risk 0.8**	January 1.	0, 0, 1, 1, 1	0.0 %
	April 1.	0, 0, $\frac{3}{4}$, 1, 1	0.1 %
	July 1.	0, 0, $\frac{1}{2}$, 1, 1	0.0 %
	October 1.	0, 0, $\frac{1}{4}$, 1, 1	- 0.1 %

Estimated parameters for the applied breast carcinoma incidence model

Table S.2 Fitted parameters for the applied incidence model.

	**Estimate**	**Std. Error**	**Pr(>|z|)**
Age	-0·1372556	0·0011491	< 2e-16
Age spline 1	0·3921366	0·0467317	< 2e-16
Age spline 2	0·6539285	0·0496109	< 2e-16
Age spline 3	1·1542253	0·0455539	< 2e-16
Age spline 4	2·1134119	0·0572616	< 2e-16
Age spline 5	3·6489167	0·0708033	< 2e-16
Age spline 6	4·8412212	0·0761691	< 2e-16
Age spline 7	5·7826226	0·1028775	< 2e-16
Age spline 8	5·7410183	0·0825007	< 2e-16
$Cohort$ spline 1	0·5559320	0·0536855	< 2e-16
$Cohort$ spline 2	0·2417406	0·1127648	0·032052
$Cohort$ spline 3	0·7539013	0·0538048	< 2e-16
$Period$ spline 1	-0·0680136	0·0261973	0·009426
$Period$ spline 2	-0·1487110	0·0716958	0·038061
$Period$ spline 3	NA	NA	NA
ht	1·1062539	0·1141012	< 2e-16
scr_1	0·4544236	0·0298591	< 2e-16
scr_2	0·1524205	0·0341511	8·08e-06
scr_{3+}	0·0830507	0·0461685	0·072041
$(scr_{3+} * \text{age})$ spline 1	0·3028698	0·0978182	0·001960
$(scr_{3+} * \text{age})$ spline 2	0·0809524	0·0513074	0·114613
scr_{pr}	-0·3964735	0·0983120	5·51e-05
$timeScr_{pr}$ spline 1	0·2836183	0·0919171	0·002031
$timeScr_{pr}$ spline 2	0·3491685	0·1280984	0·006415
$timeScr_{pr}$ spline 3	0·3752600	0·1159502	0·001211
$timeScr_{pr}$ spline 4	0·2749739	0·1114642	0·013628
$timeScr_{pr}$ spline 5	0·3030586	0·2253788	0·178734
$timeScr_{pr}$ spline 6	0·3989704	0·1491297	0·007466
$County$ 1	0·0005778	0·0214856	0·978546
$County$ 2	0·1223072	0·0183267	2·49e-11
$County$ 3	0·1499381	0·0183871	3·51e-16
$County$ 4	-0·0600419	0·0238722	0·011899
$County$ 5	-0·0553273	0·0241242	0·021823
$County$ 6	0·0592307	0·0216551	0·006235
$County$ 7	0·0378143	0·0225147	0·093048
$County$ 8	-0·0066056	0·0246959	0·789102
$County$ 9	-0·0190587	0·0302927	0·529249
$County$ 10	0·0171756	0·0257004	0·503941
$County$ 11	0·0407635	0·0201634	0·043212
$County$ 12	0·0408964	0·0190352	0·031677
$County$ 14	-0·0734422	0·0315042	0·019744
$County$ 15	0·0091890	0·0221901	0·678799
$County$ 18	-0·0861724	0·0229246	0·000171
$County$ 19	-0·1122035	0·0278636	5·65e-05
$County$ 20	-0·2431744	0·0401559	1·40e-09
$County$ 50	NA	NA	NA

County codes: https://no.wikipedia.org/wiki/Fylkesnummer#Fylkesnummer_1946–2018/2020

Calculating the proportion of breast carcinomas that are screening-detected

When calculating the frequency of screening-detected cases using the incidence model, we acquired the number of screening-detected cases for the period 2016-2019 from the Cancer Registry of Norway. These screening-detected cases included women from 49 to 71 years of age, due to the organization of the program by birth cohorts. We considered the screening-detected cases as proportion of all cases for women aged 50-69 years for the period 2016-2019, as this is the targeting age for the screening program. This might give a weakly biased estimate of the proportion of breast carcinomas that are screening-detected (68%), but the increased incidence is most prominent in the 50-69 age range, and including women 49-71 years of age would likely have biased the estimate more (Figure S.4).

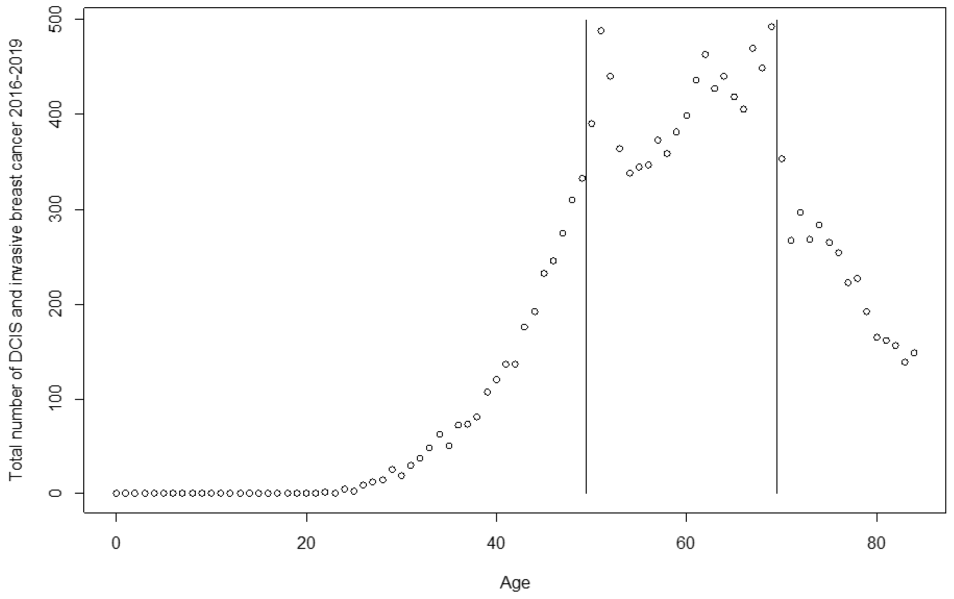

Figure S.4 The total number of first cases of breast carcinomas in Norway 2016-2019, by age.

Analysis of invasive breast cancer only

When restricting the analyses to only invasive breast cancers, the study included 59 027 cases. In the estimation of the proportion of non-progressive invasive cases related to screening (Table S.4), we applied the observed proportion of screening-detected invasive breast cancers among women 50-69 years of age in the years 2016-2019 of 63%. The analysis estimates that 21% (227/1069) of the excess cases during the screening period would have appeared as a clinical invasive breast cancer by 85 years of age in women who were not invited to screening (Table S.3). However, it is important to realize that the analysis might be somewhat biased by screening-detected DCIS cases that did not have the opportunity to develop into invasive cancers. Hence, the given estimates and related confidence intervals might be somewhat low.

Table S.3 Basis for excess cumulative incidence calculations for invasive cases only, based on estimates from the APC incidence model calculated for the 1969 birth cohort in 2019. All numbers conditional on the women being alive.

		Among 100 000 women		
Age	Relative incidence with screening program 50-69 years of age	Excess incidence	Incidence deficit	Excess cumulative incidence
50	1.462	97	-	97
51	1.462	97	-	193
52	1.098	21	-	214
53	1.098	21	-	235
54	1.030	7	-	242
55	1.054	13	-	255
56	1.077	19	-	274
57	1.099	25	-	299
58	1.121	32	-	331
59	1.140	39	-	370
60	1.158	45	-	415
61	1.173	51	-	466
62	1.185	57	-	524
63	1.195	63	-	587
64	1.203	69	-	655
65	1.209	74	-	729
66	1.213	79	-	808
67	1.215	83	-	891
68	1.216	87	-	978
69	1.216	90	-	1069
70	0.663	-	-146	923
71	0.709	-	-130	793
72	0.823	-	-81	712
73	0.890	-	-52	659
74	0.919	-	-39	620
75	0.934	-	-33	587
76	0.948	-	-27	560
77	0.958	-	-22	539

78	0.962	-	-20	518
79	0.955	-	-25	494
80	0.934	-	-36	458
81	0.907	-	-53	405
82	0.889	-	-64	341
83	0.893	-	-64	277
84	0.919	-	-50	227

Table S.4 Non-progressive invasive breast cancers, with follow-up until 85 years of age

	Proportion non-progressive cancers of screening-detected cases	Probability of a non-progressive cancer after 10 screening rounds	Number of women screened for 10 rounds per screening-detected non-progressive cancer
Invasive cases only	5·2% (-9.0,17.4)	0·2% (-0.4,0.8)	441 (-3828,4355)[a]

a The low probability of a non-progressive invasive cancer contributes to a very wide confidence interval.

References

1. Norwegian Institute of Public Health. WHO Collaborating Centre for Drug Statistics Methodology. [22 April 2023]. Available from: www.whocc.no/.
2. Duffy SW, Jonsson H, Agbaje OF, Pashayan N, Gabe R. Avoiding bias from aggregate measures of exposure. J Epidemiol Community Health. 2007;61(5):461-3.
